# A new framework for teaching scientific reasoning to students from application-oriented sciences

**DOI:** 10.1007/s13194-021-00379-0

**Published:** 2021-06-02

**Authors:** Krist Vaesen, Wybo Houkes

**Affiliations:** Philosophy & Ethics, School of Innovation Sciences, Eindhoven University of Technology, P.O. Box 513, 5600 MB Eindhoven, The Netherlands

**Keywords:** Science education, Model-based reasoning, Hypothesis-driven research, Application-oriented research, Epistemic activities, Ronald Giere

## Abstract

About three decades ago, the late Ronald Giere introduced a new framework for teaching scientific reasoning to science students. Giere’s framework presents a model-based alternative to the traditional statement approach—in which scientific inferences are reconstructed as explicit arguments, composed of (single-sentence) premises and a conclusion. Subsequent research in science education has shown that model-based approaches are particularly effective in teaching science students how to understand and evaluate scientific reasoning. One limitation of Giere’s framework, however, is that it covers only one type of scientific reasoning, namely the reasoning deployed in hypothesis-driven research practices. In this paper, we describe an extension of the framework. More specifically, we develop an additional model-based scheme that captures reasoning in application-oriented practices (which are very well represented in contemporary science). Our own teaching experience suggests that this extended framework is able to engage a wider audience than Giere’s original. With an eye on going beyond such anecdotal evidence, we invite our readers to test out the framework in their own teaching.

## Introduction

The late Ronald Giere wrote a widely used textbook, entitled *Understanding Scientific Reasoning*, meant to introduce lower-division students to scientific reasoning. Throughout its four editions, the book was designed to impart to students the ability to *understand* and *evaluate* bits of scientific reasoning, as instantiated in popular press articles, semi-professional technical reports and scholarly publications. Given this aim, the book avoids in-depth historical reflection on the philosophy of science, or on the evaluative framework it adopts. Rather, in every edition, Giere simply introduces his framework, and then moves on to how it can be used.

Giere’s framework changed over time, though. In the first (1979) and second (1984) editions of the book, it fits the traditional statement approach, which Giere traces back to Mill’s *A System of Logic* (1843). This was in line, as he reported afterwards (Giere, 2001), with what he took to be the approach in the vast majority of textbooks in logic and reasoning. The statement approach assumes that

“the evaluation of any particular bit of reasoning is done by first *reconstructing that reasoning as an explicit argument*, with premises and a conclusion, and *then examining the reconstructed argument to see if it exhibits the characteristic form of a good argument, whether deductive or inductive*” (Giere, 2001, p. 21, italics added).

The basic aim of the statement approach is to determine whether one or more statements or linguistic expressions (viz., the conclusion of an explicit argument) are true or false or, at least, to determine whether it is reasonable to take the statements to be true or false on the basis of other statements. In the third (1991) and fourth (2005) editions, Giere abandons this approach in favour of a model-based approach. This reflects a then growing concern among philosophers of science that modern scientific claims simply do not translate well into statements, leading to ill-fitting or impoverished reconstructions. For instance, the behavior of complex systems such as coupled harmonic oscillators or of randomly breeding populations of predators and prey is typically represented by mathematical models; brain processes or processes within organizations are commonly represented by diagrams; and the study of turbulence and energy systems tends to be informed by the study of scale models. Even if one were to succeed in turning these different types of models into sets of linguistic expressions, it would, according to Giere, be pointless to assess the truth of such expressions: such expressions are true, by definition, of the models, but not of the world. Also, Giere contends that, since models are abstract objects, the relationship between model and world is not one of truth, but rather one of fit. Scientists are primarily engaged in assessing the fit between models and target systems in the real world, i.e., in assessing whether their models are sufficiently similar to target systems to study the behavior of the latter by means of the former.

Giere indicates that his model-based approach better resonates with students. This matches our own experience in teaching scientific reasoning. There is also more systematic evidence for the advantages of model-based approaches. For one, there is widespread consensus among researchers that model-based reasoning more accurately describes actual scientific cognition and practice than argumentative reasoning (Clement, 2008; Gilbert et al., 1998; Halloun, 2004; Justi & Gilbert, 1999; Passmore & Stewart, 2002; Taylor et al., 2003). Cognitive scientists even have proposed that human cognition *in general* (not just scientific cognition) is best described in terms of mental modelling (Johnson-Laird, 1983, 2006; Nersessian, 2002, 2008). Furthermore, in their typical science courses, students are introduced to theories by means of models rather than arguments. Model-based approaches, thus, tap into customary modes of thinking among science students and, accordingly, appear effective in science instruction (Böttcher & Meisert, 2011; Gobert & Clement, 1999; Gobert & Pallant, 2004; Gobert, 2005; Matthews, 2007). Finally, from a more evaluative perspective, statement approaches struggle to accommodate all the information that is relevant to evaluating a piece of scientific reasoning; model-based assessments fare much better in comparison. The principal object of analysis in a statement approach is a hypothesis, which is typically expressed in a single statement. In Giere’s framework, in contrast, the object of analysis is a model. Associated with a model is not just one or more hypotheses, but also crucial background information, such as auxiliary assumptions (i.e., assumptions that are assumed to hold but that are secondary to the hypotheses under investigation) and boundary conditions (i.e., the conditions that need to be satisfied for a proper empirical test of the model and its hypotheses). Additionally, in Giere’s framework a model is explicitly evaluated relative to competing models. Here, Giere does not distinguish between different types of models: he presents a framework that is meant to apply to mathematical models, scale models, and diagrams alike, focusing on their shared role in scientific reasoning.

In Section 2, we will discuss Giere’s model-based framework in more detail, focusing on its role as an instrument to instruct students how to go about evaluating instances of scientific reasoning. In doing so, we will identify a serious limitation: the framework captures only one mode of reasoning, namely the reasoning employed in hypothesis-driven research. As teachers at a technical university, we have experienced that this makes Giere’s framework unsuitable for our particular audience. In the research practices which most of our students are educated in, hypothesis-testing is typically embedded in application-oriented epistemic activities. To capture this embedding and thus improve the usefulness to our audience, we developed an extension of Giere’s framework. Section 3 introduces this extended model-based framework for assessing application-oriented research. Section 4 discusses the wider applicability of our extended framework. Since much of contemporary science is application-oriented rather than hypothesis-driven, we submit that our framework will also benefit teachers that work outside the confines of a technical university.

## Giere’s framework

**Fig. 1:**
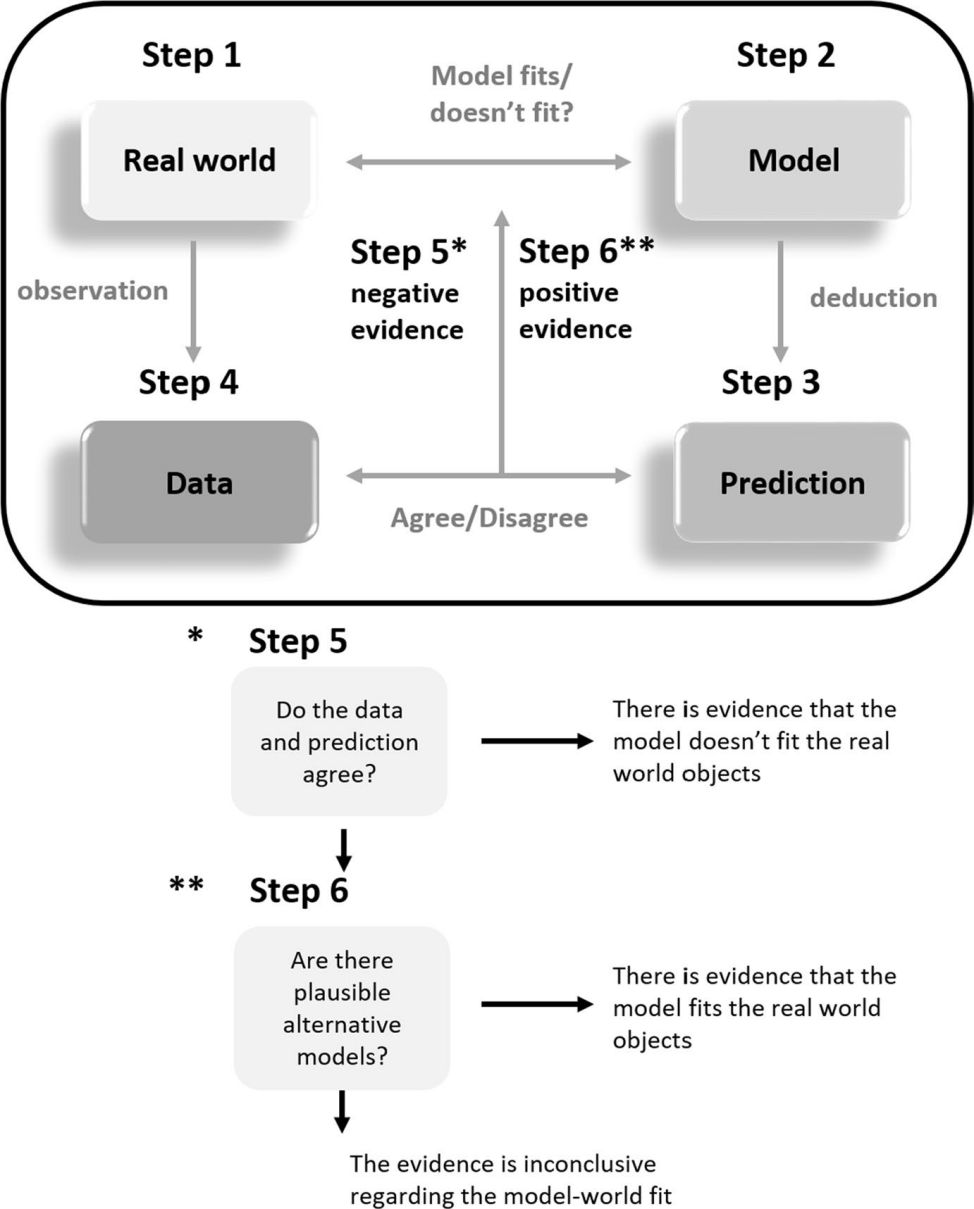
Steps in analysing hypothesis-driven approaches; Step 1—Real world: identification of the target system, i.e., the aspect of the world that is to be captured by the model. Step 2—Model: development of a model, which is to be assessed for its fit with the target system. Step 3—Prediction: deduction of predictions from the model. Step 4—Data: data collection from the target system, in order to establish the (non-)agreement between data and predictions. Step 5/6—Negative/Positive evidence: evaluation of model-target fit, based on the (non-)agreement between data and prediction

In Giere’s model-based alternative to the reconstructive statement approach, the primary purpose of observation, experimentation and scientific reasoning is to assess the fit between models and real-world target systems. Giere developed a representation of this decision process (see Fig. 1, and the caption that accompanies it) to aid students in evaluating (popular) scientific reports; and here, we will use this representation together with an example to outline his framework.

**Fig. 2:**
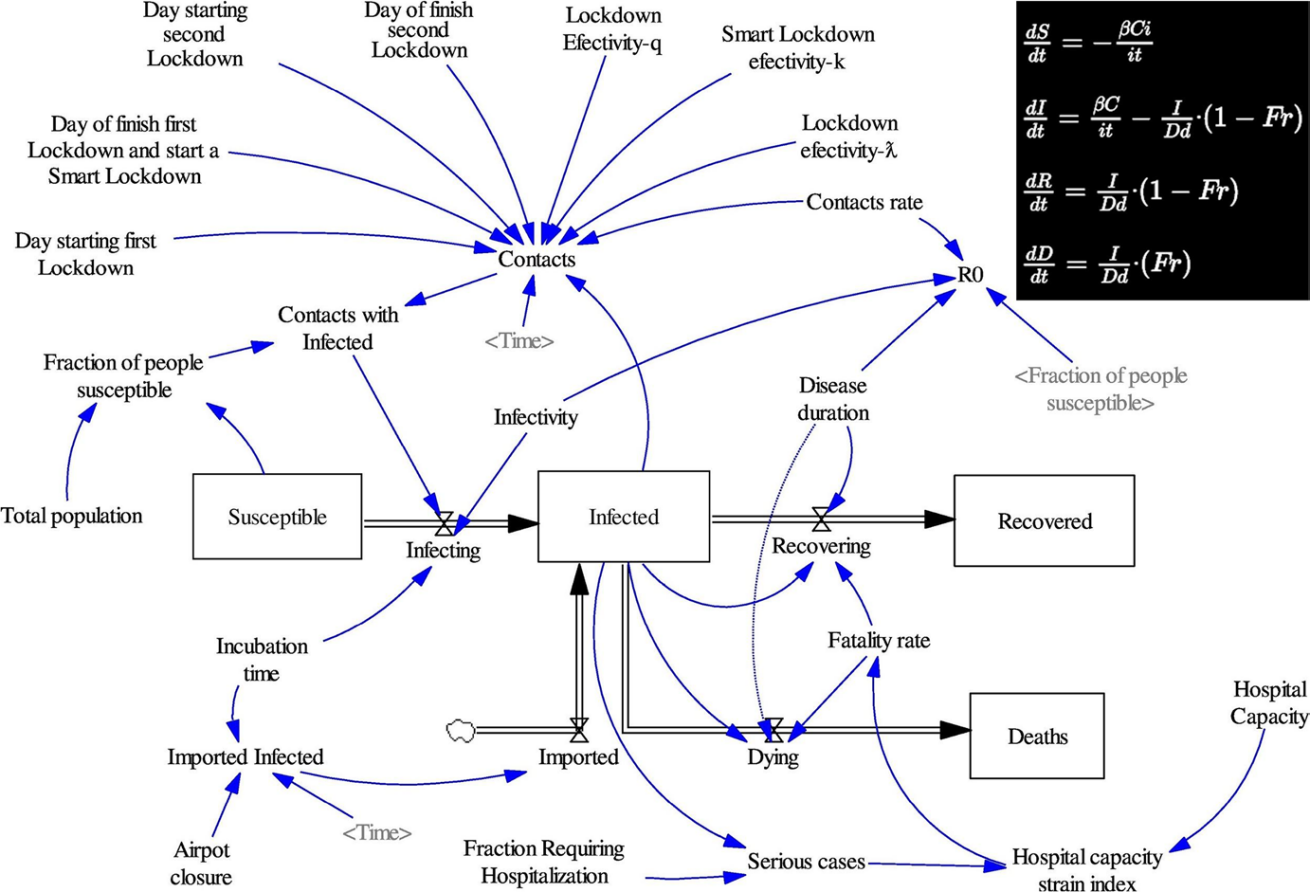
Epidemiological model of COVID-19 (taken from Vega, 2020). The graph shows the (interactions among) independent variables, and how they affect the dependent variables (“Susceptible”, “Infected”, “Recovered” and “Deaths”). The equations (top-right) express these interdependencies in a mathematical form

Consider the following example, which is accessible for a broad audience. Epidemiologists might, as per *Step 1*, identify an aspect of a real-world system that they want to better understand, for instance, the development over time of COVID-19 infections, recoveries and deaths. In *Step 2*, they develop an epidemiological model that they hope adequately captures these trends. Figure 2 presents the graphical and mathematical expressions of one such model. The graph shows various independent variables, and their interactions, and how these drive the dependent variables, viz., number of individuals susceptible to infection (S), number of infected individuals (I), number of recovered individuals (R), number of deaths (D). The mathematical expressions summarize in which ways S, I, R and D are dependent on the independent variables.

In addition to this graphical representation and mathematical expression, the model comprises auxiliary assumptions and boundary conditions. As an example of the former, the model assumes that the strain that the pandemic puts on hospitals (Hospital Capacity Strain Index) is determined by the number of serious infection cases and the hospital capacity (expressed in number of beds), where the latter is held *constant*. The model thus ignores other factors that, arguably, affect the strain on hospitals, including, lower hospital capacity due to infections among or strain on hospital personnel, the duration and frequency of pandemic waves, additional care capacity through governmental or private investment, the occurrence of an additional pandemic or epidemic, and so forth. Another auxiliary assumption is that the model population is unstructured (e.g., in terms of contact clusters, age cohorts, and so forth). As to the model’s boundary conditions, the model will fit the target system to the extent that its initial conditions reflect the target system’s initial conditions (e.g., that population sizes in the model and target system are 100,000, that the fractions of the populations that are susceptible to infection are at 13%).

**Fig. 3:**
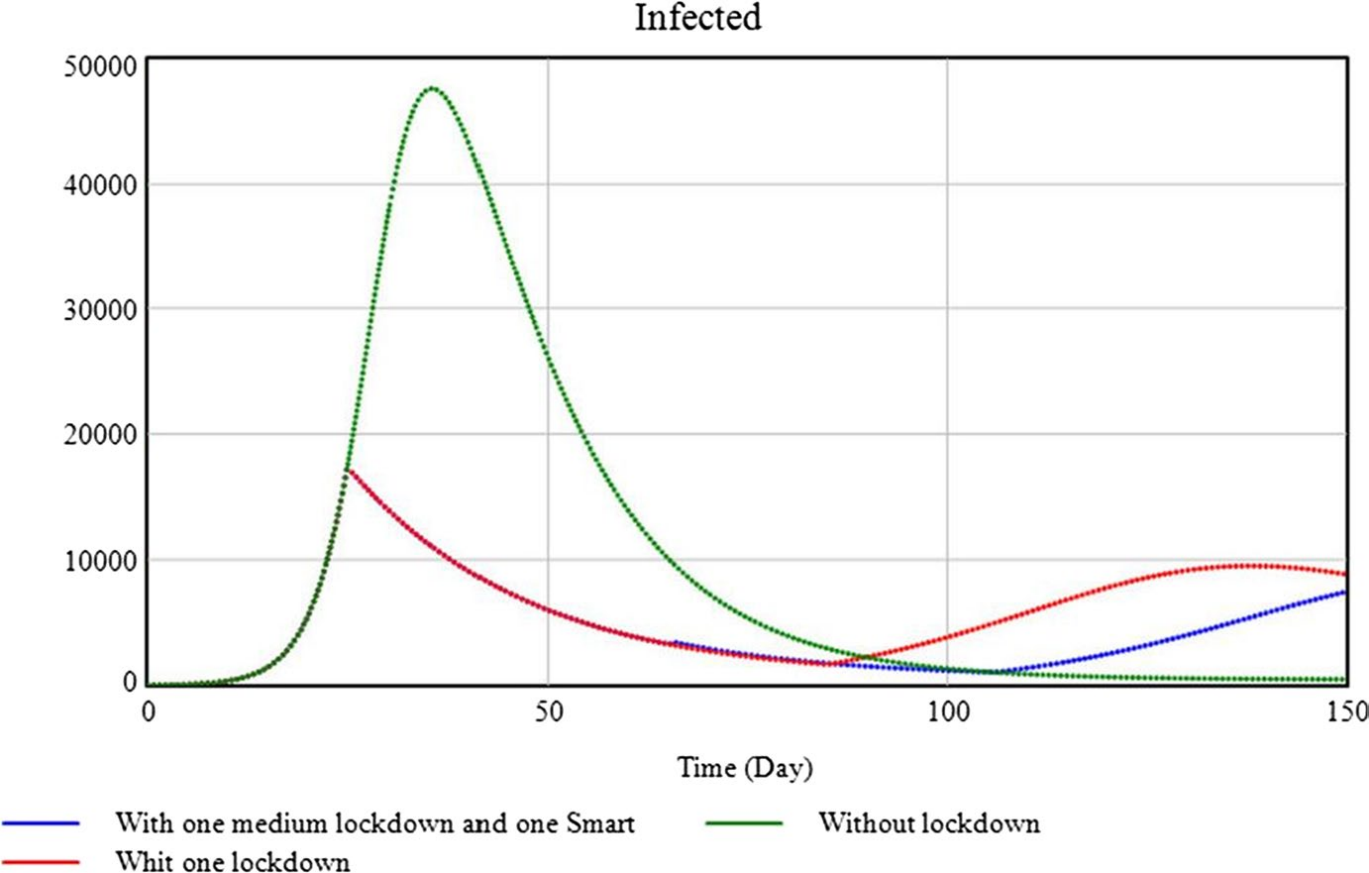
Number of infections over time, as predicted by the model of Vega (2020). The green line represents infections in a scenario without lockdown; the red and blue lines capture what would happen under different lockdown scenarios

Ultimately, the epidemiologists wish to assess the fit between the real-world target (as identified in *Step 1*) and the model (as developed in *Step 2*). In order to do so, they, in *Step 3*, derive testable hypotheses or predictions from the model. A couple of predictions are presented in Fig. 3.

Subsequently, in *Step 4*, the predictions are empirically tested, using data from the real-world target system. Here the epidemiologists might use as evidence the infection records of the first wave of COVID-19. Finally, in *Steps 5 and 6*, the agreement between this evidence and predictions (of Fig. 3) informs the epidemiologists’ evaluation of the fit between the model and real-world COVID-19 infection patterns. Negative evidence suggests a poor fit (Step 5); positive evidence, in the absence of plausible competing models, suggests a good fit (Step 6).

Having reconstructed the decision-making process of the epidemiologists along these lines, students are in a good position to evaluate it. They may formulate critical questions concerning the deduction of predictions from the model, and the inductive inferences in Step 5 and 6. Regarding the former, students should evaluate whether the predictions indeed follow from the model. Is it really the case that the prediction should hold, given the model’s auxiliary assumptions and boundary conditions? And is the prediction sufficiently surprising, precise and singular? As to the inductive inferences, to what extent are the epidemiologists’ conclusions derived in accordance with the decision tree of Steps 5 and 6? Are the epidemiologists concluding too much or too little? Do they sufficiently acknowledge remaining uncertainties, uncertainties resulting from, e.g., observational biases, low number of observations, deviations of observations from predictions, and the plausibility of competing models?

Giere’s and our own experience in using this framework confirms what research in science education has suggested about model-based approaches in general (Böttcher & Meisert, 2011; Gobert & Clement, 1999; Gobert & Pallant, 2004; Gobert, 2005; Matthews, 2007): many students find it relatively easy to internalize model-based frameworks (such as Giere’s) for evaluating reports of scientific findings. But, our own experience in teaching scientific reasoning to students in the specific context of a technical university indicates Giere’s framework doesn’t cater to everyone: for students from some programs (e.g., chemistry), internalizing the framework appears easier than for students from other programs (e.g., mechanical engineering); and in explaining the framework, we find it easier to evaluate relevant examples from one field of inquiry than from others.

This differentiation in comprehensibility of the framework brings to mind a conventional distinction between ‘fundamental’ and ‘applied’ fields of inquiry, where it is maintained that the former produce most of the theoretical knowledge that is utilized for practical problem-solving in the latter (e.g., Bunge, 1966). Since pitching this distinction at the level of fields or disciplines seems unsustainable (following criticisms reviewed in, e.g., Kant & Kerr, 2019 and Houkes & Meijers, 2021), we opt for a differentiation at the level of research practices instead.

This differentiation builds on Chang’s (2011, 2012) practice-oriented view, which distinguishes several hierarchically ordered levels of analysis: mental and physical *operations*, which constitute *epistemic activities* that in turn make up a *research practice*. Here, *research practices* are characterized by their aim, while *epistemic activities* are rule-governed, routinized sets of *operations* that contribute to knowledge production in light of these aims. Lavoisier’s revolutionary way of doing chemistry, for example, can be understood as an innovative practice in its time, constituted by activities such as collecting gases, classifying compounds, and measuring weights, which comprise various operations.

In line with Chang’s practice-oriented analysis, hypothesis testing may be taken as an epistemic activity that is more central to some research practices—such as the episode from epidemiology that was reconstructed earlier in this section—than to others. Some practices are aimed at generating precisely the theoretical knowledge that may be gained through systematic hypothesis-testing; in other practices, however, the results of hypothesis testing are instrumental to more encompassing aims. Giere’s original framework may fit the mental model of students who have been primarily exposed to or educated in the former type of practices; but it is at best an incomplete fit for the latter, more encompassing type of practice. This diagnosis suggests a natural solution: to capture these more encompassing practices, we need to extend Giere’s framework.

## A framework for assessing application-oriented research

### Reconstruction of application-oriented scientific reasoning

Research in the engineering sciences has received only limited attention from philosophers of science. Conventionally, it is understood as research that applies scientific methods and/or theories to the attainment of practical goals (Bunge, 1966). Consequently, also in the self-understanding and -presentation of many practitioners, these fields of inquiry go by labels such as ‘instrumental’ or ‘applied’ research, or are characterized in terms of ‘design’ rather than research. However, in-depth analyses of historical and contemporary episodes (e.g., Constant, 1999; Kroes, 1992; Vincenti, 1990) reveal that they involve more than merely deriving solutions to specific practical problems from fundamental theories. In particular, they can be understood as knowledge-producing activities in their own right. Some have claimed that application-oriented research involves a special type of ‘designerly’ or ‘engineering’ knowledge (as in, e.g., Cross, 1982). This knowledge has been characterized as ‘prescriptive’ (Meijers & Kroes, 2013), as consisting of ‘technical norms’ (Niiniluoto, 1993), or ‘technological rules’ (Houkes & Meijers, 2021)—but it seems fair to say that these characterizations require further specification, in their content, scope and impact on differentiating application-oriented research (see, e.g., Kant & Kerr, 2019).

For our present purposes, we only need to assume that practices in the engineering sciences, in which most of our students are trained, involve epistemic activities such as hypothesis testing and are therefore genuinely knowledge-producing. Furthermore, we submit that the knowledge thus produced often has a wider scope than specific, ‘local’ practical problems (e.g., Adam et al. 2006; Wilholt, 2006), although they might be strongly geared towards solving such problems. Given this, we label such practices ‘application-oriented’. We submit that fields of inquiry such as mechanical engineering or fusion research frequently involve application-oriented practices, without thereby expressing commitment to any of the characterizations mentioned above. Still, the framework presented below is compatible with these characterizations: it can be supplemented with (suitably developed) analyses of, e.g., technical norms or prescriptive knowledge in application-oriented practices.

**Fig. 4:**
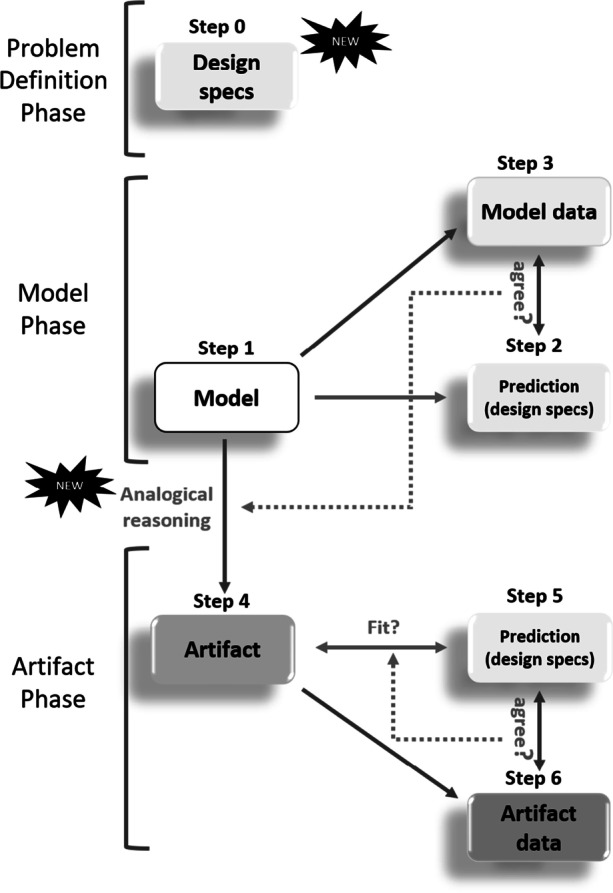
Steps in analysing application-oriented research. Problem Definition Phase: Step 0—Design specs: definition of the design specifications the artifact has to meet. Model Phase: Step 1—Model: development of model that acts as a stand-in for the artifact to be produced. Step 2—Model: derivation of predictions from the model, where predictions align with the design specs identified in Step 0. Step 3—Model data: collection of model data, and assessment of the (non-)agreement between model data and predictions. In case of agreement, and in case of reasonable analogy between model and artifact, Artifact Phase starts: Step 4—Artifact: developing artifact based on the model. Step 5: Deduction of predictions from the artifact, where predictions are identical to design specs of Step 0. Step 6—Artifact data: Collection of artifact data, and assessment of (non-)agreement between artifact data and predictions/design specs. The “New” symbols refer to procedures that are not shared with hypothesis-driven approaches

Figure 4 represents our application-oriented framework. Whereas Giere’s framework starts with a real-world phenomenon, which the researcher then wishes to capture with a suitable model, application-oriented approaches typically start with a model, which serves as a stand-in for a not-yet-existent target system (e.g., a software package, a novel protein). Or, whereas in hypothesis-driven approaches models are supposed to be descriptive of the world and to deliver theoretical knowledge, in application-oriented research, models intend to describe how the world might or should be and to yield practical knowledge. Our general assumption is that, in the epistemic activities captured by the application-oriented framework, researchers do not, without prior study, start building their real-world target system (or artifact for short). Rather, they first develop a model of the artifact (e.g., a blueprint, scale model, or computer model) and study the behavior of the model (*Model Phase* in Fig. 4). Only if the model behaves as it should, the researcher takes the next step of actually producing and testing (*Artifact Phase* in Fig. 4) the artifact for which the model served as a stand-in.

As can be seen in Fig. 4, application-oriented research in fact involves a phase prior to model-building, denoted by “Problem definition phase”. In this phase (*Step 0*), the design specs are determined, i.e., the properties or dispositions that the artifact ultimately ought to exhibit (e.g., the intended cost, functionalities and efficiency of a software program; the intended structure of a protein).

The purpose of the *Model Phase* is developing one or more *models* that meet the researcher’s predefined specs. To the extent they do, and to the extent the models are an adequate analogue for the artifact yet to be built (see Section 3.2), the researcher moves to the *Artifact Phase*. Here one goes through the same building and testing cycle as in the *Model Phase*, but this time the object of analysis is the *artifact* rather than the model. Frameworks in design methodology, such as the ‘basic design cycle’ (Roozenburg & Eekels, 1995) and the ‘Function-Behavior-Structure’ model (Gero, 1990) also represent an iterative process of determining and satisfying design specs, but without bringing out the role of model-building and thus also without explicitly distinguishing the *Model Phase* and *Artifact Phase*.

Each of these cycles bears large similarity with the cycle in Giere’s framework. In the *Model Phase*, a model is developed (*Step 1*) from which various predictions are derived (*Step 2*). Arguably, the most salient predictions are those that pertain to the design specs identified in *Step 0*, i.e., predictions concerning the extent to which the model will satisfy these specs. In order to assess this, one collects relevant data from the model (*Step 3*), and evaluates whether the data agree with the predictions (i.e., design specs). If they don’t, one might reiterate the cycle; if they do, the artifact is built based on the model (*Step 4*).

At *Step 4*, one enters the *Artifact Stage*, characterized by a similar cycle: the artifact is produced (*Step 4*); one formulates predictions about the artifact, viz., whether it exhibits the desired design specs (*Step 5)*; and one collects data that allow one to test these predictions of (*Step 6*). In case the data agree with the design specs, the artifact is accepted; otherwise, it is adjusted or steps 1–5 are reiterated. Note that the design specs of the model (*Step 2*) and the artifact (*Step 5*) might be quantitatively or qualitatively different. While the latter might simply be taken from *Step 0*, the former should fit the specific context of the model world.

**Fig. 5:**
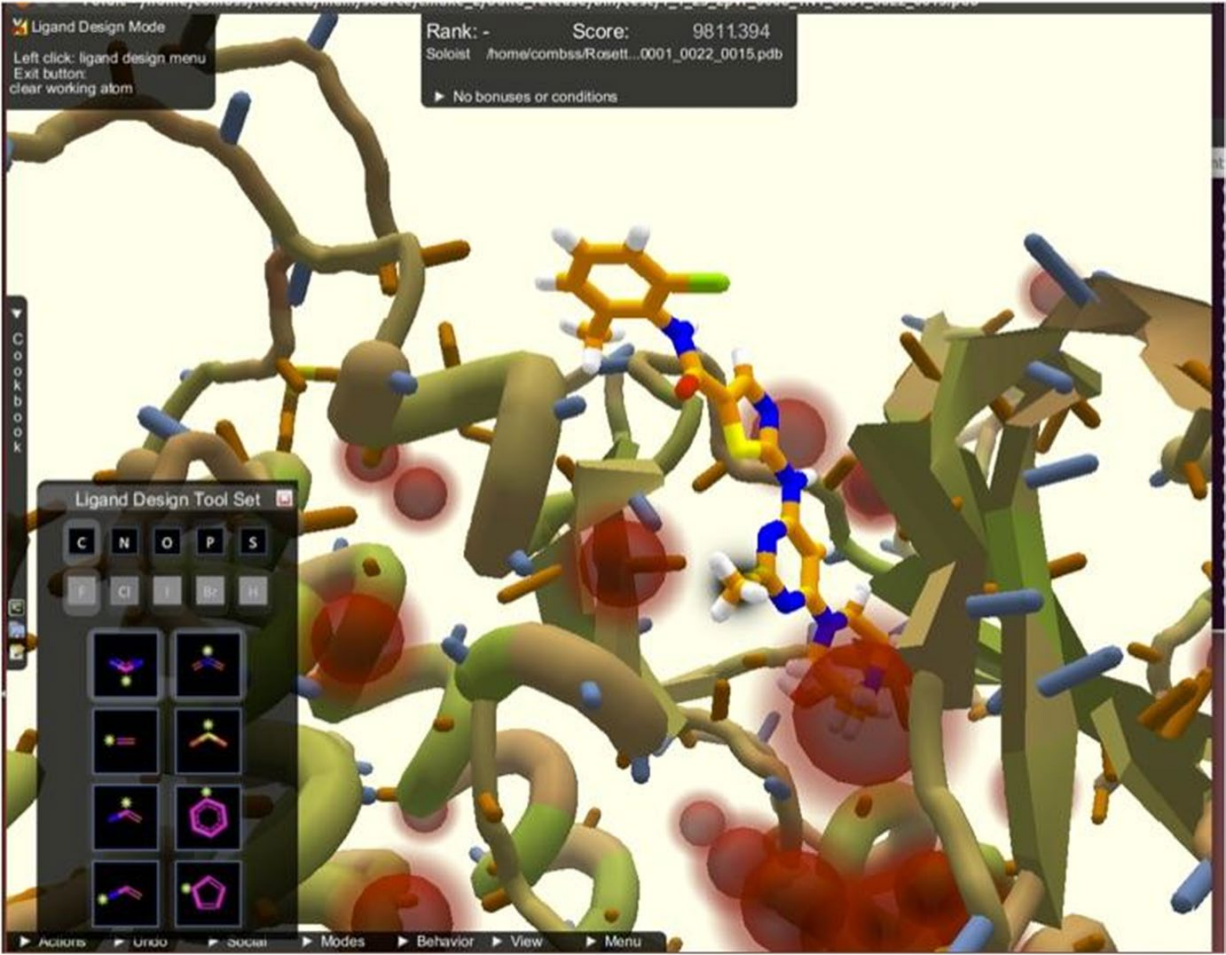
A computer model for designing new proteins

To illustrate the application-oriented framework, consider another example from the biomedical sciences, one that also pertains to COVID-19. Let us assume that a researcher’s task is to design a novel protein that is able to bind to SARS-Cov-2. Given the known structure of the binding configuration of the virus, one can define, in the *Problem Definition Phase*, the structure that the new protein ought to have and the types of (energetic) conditions under which the protein needs to remain stable (Step 0). During the *Model Phase*, in Step 1, a computer model of a candidate protein is developed (see Fig. 5). Next, in Step 2, some testable predictions are derived concerning the candidate protein’s structure and stability. Tests of its structure and stability rely on model data (Step 3). Regarding stability, for instance, the researcher/computer needs to calculate the protein’s thermodynamic free energy; a configuration that has low thermodynamic free energy is more likely to effectively exhibit the requisite structure than a configuration that has high thermodynamic free energy.

In case there is agreement between model data and model predictions, and the analogy between model and artifact (real-world protein) is adequate (see below), the researcher moves to the *Artifact Phase*, and will actually produce the protein (Step 4), typically by means of gene manipulation techniques. Step (5) and (6) then carry out a test of the real-world protein’s compliance with the design specs (e.g., structure, stability under real-life conditions, etc.).

### Evaluation of application-oriented scientific reasoning

Part of the value of a reconstruction along the lines of Fig. 4 lies in the critical questions to which it gives rise. It does so at four levels: the *Problem Definition Phase*, the *Model Phase*, the *Artifact Phase*, and at the level of the analogical inference connecting these last two phases. The evaluation of the *Model Phase* and the *Artifact Phase* are virtually identical to the evaluation involved in Giere’s hypothesis-driven framework; genuinely new evaluative steps (as indicated in Fig. 4) pertain to the *Problem Definition Phase* and the analogical inference that connects the *Model* and the *Artifact Phases*.

#### Problem definition phase (new evaluative step)

An application-oriented approach might fail as an epistemic activity well before any model or artifact is built. There are plenty of frameworks that may be used to judge the rationality of the researcher’s design specs. The researcher’s intended design specs might be—in terms of the well-known SMART-framework—insufficiently Specific, Measurable, Acceptable, Realistic or Time-related. Alternatively, students could assess the design specs along the lines of the evaluative framework of Edvardsson and Hansson (2005). This framework resembles the SMART framework, but is different in some of its details. According to it, design specs are rational, i.e., achievement-inducing, just in case they meet four criteria: precision, evaluability, approachability and motivity. Precision and evaluability are very similar to, respectively, the SMART criteria Specific and Measurable; and Approachability is a combination of SMART’s Realistic and Time-Related. Motivity, finally, refers to the degree to which a goal/design spec induces commitment in those involved in reaching it. Decision theory and the work of Millgram and Thagard (1996) suggest another evaluative criterion: the (in)coherence among design specs. Teachers might find still other frameworks useful; but in any case, the merit of our proposed teaching approach is that it forces students to reflect on the rationality of the problem definition phase.

#### Model phase

Given the strong parallel between Giere’s framework and our *Model Phase,* the latter can largely be evaluated according to Giere’s evaluation principles. Students first should determine whether the prediction of *Step 2* indeed reasonably follows from the model. Is it feasible for the model to meet the design specs, given its auxiliary assumptions and boundary conditions? For instance, the researcher in the example might choose to redesign an existing protein rather than to model a protein from scratch. Accordingly, is it reasonable to think that the existing protein, given its structure and other properties (auxiliary assumptions), can ever be redesigned in such a way that it will behave as desired? Further, the prediction has to be assessed in terms of surprisingness, i.e., the degree to which the prediction goes beyond common sense; precision, i.e., the degree to which the prediction is specific rather than vague; and singularity, i.e., the degree to which the prediction is not a conjunction of predictions.

Next, students are to evaluate the (lack of) agreement between predicted design specs (*Step 2*) and the data from *Step 3*. Such evaluation involves the assessment of the quality of the data (e.g., number of observations, (in)variance of data across different conditions, deviations of data from predicted values), and informs the decision as to build a new model (in case of non-agreement) or to move to the *Artifact Phase* (in case of agreement). The latter decision is informed also by analogical reasoning.

#### Analogical reasoning (new evaluative step)

The model only forms a proper basis for the development of the artifact if the two are sufficiently similar in relevant respects. This gives rise to assessing an analogical inference of the following form: one observes that, in virtue of model’s properties *a,b,c*, the model meets design spec *x*; accordingly, an artifact that, analogously to the model, has properties *a,b,c*, will probably also meet design spec *x*.

The strength of this inference depends on the extent and relevance of the similarities and dissimilarities between model and artifact. Students should first identify all relevant similarities and dissimilarities, where the criteria of relevance are set by the design specs. For instance, in order to justify the translation from model to real-world protein, a comparison between the surrounding environment of the model protein and the surrounding environment of the real-world protein is clearly relevant. Similarity in color, in contrast, says nothing about the real-world protein’s stability. Furthermore, students must assess the degree and number of these relevant similarities, and do the same for relevant *dis*similarities.

Finally, students need to identify other, independent models (e.g., scale model, other computer models) of the artifact to be produced, and assess the relevant (dis)similarities between these models and the artifact. It would strengthen the analogical inference if such existing models point in the same direction as the model under study. Conversely, their confidence in the analogical inference should decrease when other models that, in the relevant ways, are similar to the artifact do not satisfy the design specs.

#### Artifact phase

Assessment of the *Artifact Phase* largely follows the same procedure as the procedure described under the bullet *Model Phase*. Only the goals of the phases are different. Whereas the goal of the *Model Phase* is producing information that is relevant for further steps in the research process, the final step of the *Artifact Phase* simply ends the entire exercise. Ideally, the latter phase results in an artifact that meets the design specs identified in *Step 0*.

## Conclusion and discussion

We familiarize all our science students with *both* frameworks, for the simple reason that students typically encounter both types of research in their curriculum and later professional career. After all, parts of hypothesis-driven research are in fact application-oriented (e.g., design of experiments and equipment, tailor-making computer code), and parts of application-oriented research are hypothesis-driven (e.g., incorporation of hypothesis-driven theories into the design exercise). Further, in many disciplines both approaches peacefully coexist. As our examples of COVID-19 research show, the biomedical sciences comprise different research practices and corresponding epistemic activities; some of these are more hypothesis-driven, others more application-oriented.

**Fig. 6:**
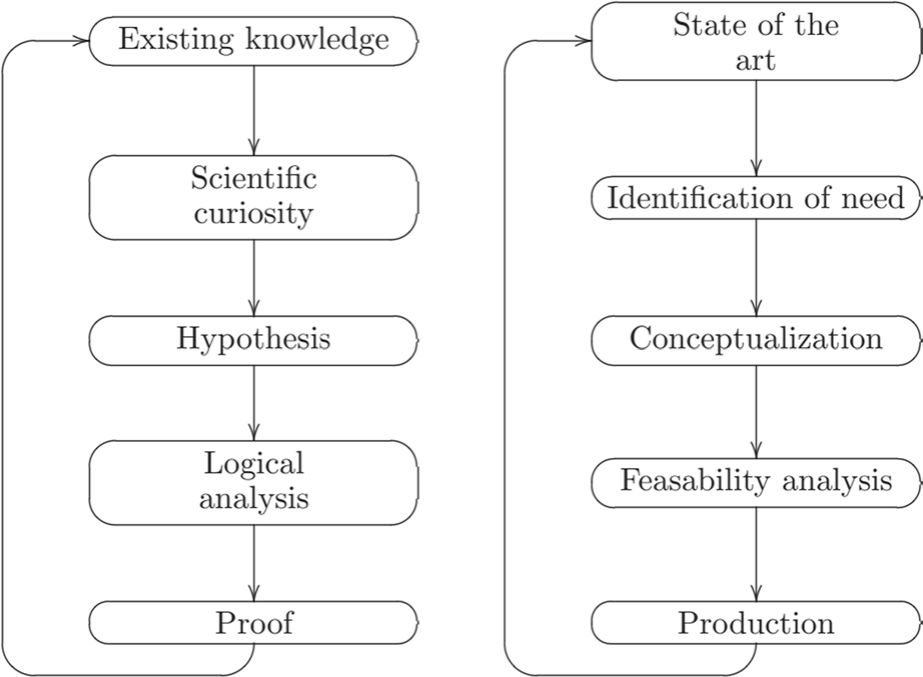
Hill’s (1970) comparison of scientific reasoning (left) and reasoning in engineering sciences (Figure taken from Hughes, 2009)

Teaching the two frameworks together is also very efficient. There are a number of elements that recur in both approaches; learning about one approach facilitates learning about the other. Given such similarities, it is also easier for students to see and appreciate the crucial differences between the frameworks. These features are a great plus. Standard reconstructions of the research processes in the sciences and the engineering sciences give the impression that there is only limited overlap between the two (see, e.g., Fig. 6). We have seen, however, that application-oriented research relies on scientific tools, methods and modes of reasoning. Our framework makes this explicit.

On a final note, our new framework has been mainly developed in response to perceived shortcomings for students at a technical university. There are, however, reasons to suppose that it could be deployed in courses for students from a wide range of disciplines. It has been noted by many that research in many disciplines has undergone a shift away from ‘fundamental’ issues to more ‘applied’ ones; that research efforts have become more interdisciplinary in response to financial and societal incentives; and that new fields of inquiry (e.g., biomedical research, education research, social and economic policy research, management science, intervention research, experimental design research), tend to be oriented towards particular areas of application. Many different interpretations have been given, for instance in terms of changing ‘modes’ of knowledge production (Gibbons et al., 1994); a ‘triple helix’ of private, public and academic partners (Etzkowitz & Leydesdorff, 2000); or a ‘commodification’ of academic research (Radder, 2010). So if we accept that it is taking place in some form, it entails that an increasing number of students would in the course of their educational programs be primarily exposed to application-oriented research practices and epistemic activities. Thus, if our experiences would generalize, philosophy of science teachers might well find that our extended version of Giere’s model-based framework is more comprehensible and useful for ever more students than Giere’s original version, let alone a statement-based approach. Surely, whether our experiences in fact generalize remains to be seen. In building on Giere's original framework, we have provisionally adopted his (implicit) assumption that different types of models – e.g., mathematical models, scale models, and diagrams – play sufficiently similar roles in scientific reasoning to be treated alike. A more differentiated approach may well be called for. Likewise, we have supposed that our framework for reconstructing application-oriented research is compatible with different proposals regarding the distinctive knowledge generated by such research (e,g., in the form of technical norms or other prescriptive knowledge). This supposition may well be incorrect, i.e., may turn out to gloss over distinctive features that would allow students from some programs to gain insight into research activities in their chosen disciplines. Such shortcomings can, however, be best identified in practice rather than discussed in the abstract. We therefore invite other teachers to more systematically study the merits and downsides of our application-oriented version of Giere's model-based framework.

## Data Availability

Not applicable.
